# Advancing healthcare allocation and prevention of disability: the role of disease-based predictive model for disability in aging adults

**DOI:** 10.1186/s12877-025-06457-9

**Published:** 2025-10-21

**Authors:** Yi-Chun Lin, Yu-Ning Chien, Wan-Chun Yang, C.-Y. Yvonne Lai, Kwang-Hwa Chang, Shih-Wei Huang, Tsan-Hon Liou, Hung-Yi Chiou

**Affiliations:** 1 School of Public Health, Taipei Medical University, Taipei, Taiwan; 2 Department of Health and Welfare, University of Taipei, Taipei, Taiwan; 3 Institute of Population Health Sciences, National Health Research Institutes, Miaoli, Taiwan; 4 Department of Physical Medicine and Rehabilitation, Wan Fang Hospital, Taipei Medical University, Taipei, Taiwan; 5 Department of Physical Medicine and Rehabilitation, Shuang Ho Hospital, Taipei Medical University, New Taipei City, Taiwan

**Keywords:** Disability prevention, Predictive modeling, Long-Term care, Risk assessment

## Abstract

**Background:**

Disability in the aging population was a major public health challenge for aging nations, imposing a significant burden on healthcare resources. Accurate disability prediction models were essential for efficiently allocating long-term care resources and preventing disability. This study utilized healthcare claims data to construct a disease-based disability risk prediction model that identified high-risk disability groups and diseases with significant impacts on disability. The model informed the formulation of prevention strategies and resource allocation.

**Methods:**

This study adopted the Long-Term Care Database to define disability in the aging population and utilized the National Health Insurance Research Database to construct disability-related disease variables. Five machine learning models were employed to build the disability risk prediction model. The model assessed the risk of disability for each elderly adult based on disease status and identified individuals with disabilities in the aging population. Additionally, the Shapley Additive Explanation method was employed to analyze the extent to which diseases impacted disability and to identify illnesses that significantly influenced disability.

**Results:**

The study revealed that among all the algorithms tested, the XGBoost algorithm exhibited the strongest predictive power. Its AUC was 0.867, and its balanced accuracy was 0.795. Based on the feature importance ranking generated by the disability risk prediction model, chronic conditions, including renal failure, dementia, cerebral vascular obstruction and stenosis, and hypertension, were found to be significantly associated with disability.

**Conclusions:**

The disability risk prediction model provided a real-time digital prediction mechanism to identify high-risk groups of disability among elderly adults, serving as a valuable decision-making tool for disability prevention and the allocation of medical care resources. Developing prevention and treatment strategies targeting the chronic diseases identified as significant contributors to disability by the predictive model might lead to more effective prevention of disability in elderly adults.

**Supplementary Information:**

The online version contains supplementary material available at 10.1186/s12877-025-06457-9.

## Introduction

The escalating prevalence of elderly persons with disabilities strained healthcare systems. With the demographic shift towards an older population, the financial demands stemming from these disabilities surged, creating a pressing issue for countries with an aging populace [1–4]. Thus, formulating a predictive model for disability trends in seniors and strategizing healthcare and social service distribution to mitigate this growing concern was imperative for public health [2, 5–7].

Elderly persons with disabilities primarily stemmed from reduced physical and cognitive abilities, which impacted their daily activities [8–10]. Physical frailty, cognitive deterioration, chronic conditions, and multimorbidity significantly contributed to these disabilities [8, 11–13]. Research indicated that 51% of disabilities and reduced quality of life in this demographic were due to age-related diseases [4]. Globally, 23% of the disease burden was Linked to illnesses common in those aged 60 and over [1]. Notably, these diseases more frequently resulted in disabilities than in fatalities, and the financial demands of long-term care surpassed those of disease treatment, further burdening caregiving resources in society [4].

Effective care resource planning for elderly persons with disabilities hinged on precise predictive models identifying high-risk groups [2, 5]. Focusing on these groups with targeted, effective prevention and care resources was crucial to diminishing overall care costs [5].

Existing disability prediction models commonly incorporated socioeconomic status, social class, and self-reported health status as predictive variables. Comorbidities were also recognized as important predictors of disability [14–17]. Some studies further utilized health insurance claims data and electronic health records to highlight chronic diseases as principal predictors for forecasting frailty and disability among elderly adults over extended periods [18–22]. Evidence indicated that chronic conditions such as arthritis, diabetes, cardiovascular disease, osteoporosis, pulmonary disease, hypertension, musculoskeletal disorders, and stroke were strongly associated with an increased risk of disability. These findings aligned with previous clinical observations suggesting that disability frequently resulted from the coexistence of multiple chronic diseases [23–27]. Notably, elderly adults with two or more chronic conditions were reported to face a five- to six-fold higher risk of physical disability compared to those without such conditions [28, 29]. As the prevalence of multimorbidity continued to rise with aging populations, chronic disease-related variables demonstrated superior predictive value for disability.

This study employed national healthcare datasets to identify key predictors of disability-related conditions and used machine learning to create a disease-based disability risk prediction model for the elderly. The predictive model demonstrating the best performance was used to identify high-risk groups for disability and to support optimal care resource allocation. Additionally, its insights on disease rankings contributing to disability were considered vital for developing preventive strategies for the aging population.

## Study design, data and methods

### Study design

**Fig. 1:**
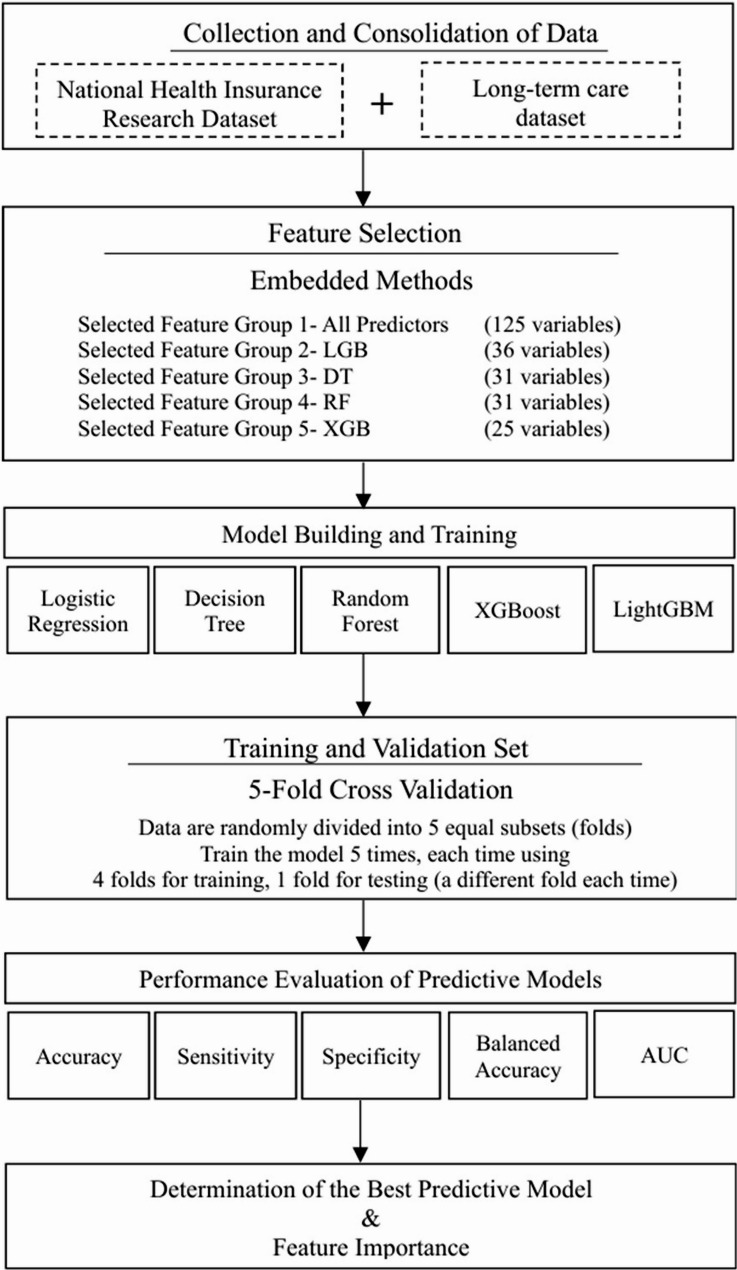
Flow chart of the study design for constructing an elderly disability risk prediction model. The abbreviation* LGB* stands for Light gradient-boosting machine (LightGBM), *DT* refers to the Decision Tree Model, *RF* refers to the Random Forest Model, and *XGB* stands for eXtreme Gradient Boosting (XGBoost) model

This research presented a methodology for developing a predictive model of disability in the elderly, as depicted in Fig. 1. The process began with integrating the National Health Insurance Research Database (NHIRD) and Long-Term Care Database (LTCD) in Taiwan to form disease predictors and patient demographics for the model. Relevant variables were identified via embedded feature selection techniques. The dataset was divided into an 80% training set and a 20% testing set, with data imbalance adjustments applied to improve prediction accuracy. Model validation employed a 5-fold cross-validation approach. Model performance was evaluated using metrics such as sensitivity, specificity, balanced accuracy, and the average Area Under the Receiver Operating Characteristic Curve (AUC). The most accurate model was selected, and the Shapley Additive Explanation (SHAP) method was employed to pinpoint key disease-related variables, thereby aiding policymakers in crafting efficient intervention strategies.

### Data

In this research, Taiwan’s National Health Insurance Research Database (NHIRD) and Long-Term Care Database (LTCD) were harnessed to construct a disability risk prediction model for the aging demographic. The LTCD, a vast national repository, encompassed data on long-term care types and levels, disability status, and associated information, facilitating the identification of Taiwan’s disabled population [30]. The NHIRD, a comprehensive nationwide longitudinal database covering 99% of citizens, provided extensive medical claim details, including diagnosis history, medication usage, hospitalization, and surgical records. These data were instrumental in developing an exhaustive set of disease-predictive variables for the aging population.

### Study population

This research analyzed a cohort of 3,949,716 individuals aged 65 and above from the NHIRD between 2017 and 2020. Within this group, 433,623 seniors required long-term care due to disabilities, constituting approximately 11% of the study population, while the remaining 3,516,093 were classified as non-disabled.

As detailed in Table 1, the disabled group was generally older, had a higher proportion of females, and exhibited a greater incidence of comorbidities compared to their non-disabled counterparts. These demographic and health distinctions underscored a strong correlation between comorbidities and disability, providing critical parameters for machine learning models to accurately identify disability risks.

**Table 1:** Baseline characteristics of elderly adults with and without disabilities

	Elderly adults with disabilities (*N* = 3,516,093)	Elderly adults without disabilities (*N* = 433,623)	*P*-value ^#^
Sex, n(%)			< 0.001
Female	1,890,896 (53.78%)	251,043 (57.89%)	
Male	1,625,197 (46.22%)	182,580 (42.11%)	
Age, n(%)			< 0.001
65–70	1,628,777 (46.32%)	59,691 (13.77%)	
71–75	748,159 (21.28%)	57,418 (13.24%)	
76–80	533,719 (15.18%)	88,536 (20.42%)	
81–85	340,763 (9.69%)	98,578 (22.73%)	
> 85	264,675 (7.53%)	129,400 (29.84%)	
Mean (SD)	73.26 (7.21)	80.67 (8.02)	< 0.001
Comorbidities, n(%) *			
Renal failure	288,019 (8.19%)	100,891 (23.27%)	< 0.001
Senile and presenile organic psychotic conditions	162,407 (4.62%)	97,971 (22.59%)	< 0.001
Lower limbs Fractures	217,790 (6.19%)	73,589 (16.97%)	< 0.001
Hypertension disease	2,036,182 (57.91%)	343,607 (79.24%)	< 0.001
Occlusion and stenosis of cerebral arteries	210,964 (6.00%)	98,077 (22.62%)	< 0.001
Diabetes mellitus	1,061,443 (30.19%)	181,903 (41.95%)	< 0.001
Pneumonia	185,476 (5.28%)	86,230 (19.89%)	< 0.001
Late effects of cerebrovascular disease	105,127 (2.99%)	64,898 (14.97%)	< 0.001
Fracture of vertebral column without spinal cord injury	132,257 (3.76%)	50,537 (11.65%)	< 0.001
Heart failure	164,585 (4.68%)	70,057 (16.16%)	< 0.001
Parkinson’s disease	79,716 (2.27%)	45,633 (10.52%)	< 0.001
Malignant neoplasm of others †	66,056 (1.88%)	23,897 (5.51%)	< 0.001
Mental disorders ‡	913,136 (25.97%)	182,059 (41.99%)	< 0.001
Intracranial hemorrhage	65,697 (1.87%)	36,516 (8.42%)	< 0.001
Other organic psychotic conditions (chronic)	173,117 (4.92%)	91,652 (21.14%)	< 0.001
Malignant neoplasm of digestive organs and peritoneum	118,270 (3.36%)	30,093 (6.94%)	< 0.001
Fracture of vertebral column with spinal cord injury	131,722 (3.75%)	40,976 (9.45%)	< 0.001
Hemiplegia and hemiparesis	25,570 (0.73%)	22,921 (5.29%)	< 0.001

* Comorbidities are the top 18 important diseases among the 123 diseases obtained from the XGBoost model

† Malignant neoplasm of others include eye, brain, thyroid gland, unspecified parts of nervous system, other endocrine glands, secondary malignant neoplasm

‡ Mental disorders include neurotic disorders, personality disorders, other psychotic mental disorder

# *P*-values were calculated using t-tests for continuous variables (e.g., age) and chi-squared tests for categorical variables (e.g., sex and comorbidities). All tests were two-tailed, and a *p*-value of less than 0.05 was considered statistically significant

### Primary outcome measure

The long-term care case-mix system (CMS) was the official standard used by Taiwan’s long-term care authorities to evaluate an individual’s disability status and determine eligibility for service Subsidies. The CMS was a composite scoring system that integrated 11 dimensions, including activities of daily living (ADL), instrumental activities of daily living (IADL), emotional and behavioral problems, mental disorders, communication ability, cognitive functioning, continence, mobility, self-care ability, health-related conditions, and complex medical care needs [31]. Each domain was assessed through a structured evaluation conducted by trained social workers, who scored each item based on the severity of impairment. The aggregated scores generated a CMS level ranging from 0 (no disability) to 8 (extremely severe disability), with higher levels indicating greater disability severity.

In this study, individuals with a CMS level of 2 or higher were considered to have disabilities, in accordance with the eligibility criteria under Taiwan’s Long-Term Care 2.0 program [32]. Therefore, the disability group was defined as older adults enrolled in the Long-Term Care Database (LTCD) who had a CMS level equal to or greater than 2.

Importantly, CMS assessments were more comprehensive than relying solely on ADL or IADL scores, as they incorporated multiple functional and clinical domains. The CMS score not only reflected the overall disability status but also determined the corresponding financial subsidies and types of services provided to eligible individuals. Supplementary Material Table S1 presented the possible ADL/IADL scores, associated service categories, and subsidy amounts corresponding to each CMS level.

### Disease-Based predictive variables

Disability prediction features were derived from age, gender, and 123 disease conditions related to disability in the study group, with extensive disease details provided in Supplementary Material Table S2. The number of medical records pertaining to these disability-associated diseases over the three years preceding the disability onset was computed. A disease was confirmed for inclusion in the study if it had been recorded three or more times.

These 123 disease conditions related to disability were categorized into 23 groups: hypertension, diabetes, bone disease, visual impairment, cerebrovascular disease, transient ischemic attack, coronary artery disease, atrial fibrillation/other arrhythmias, cancer, respiratory, digestive, and genitourinary system diseases, dementia, mental illness, autism, intellectual disability, cerebral palsy, Parkinson’s disease, spinal cord injury, motor neuron disease, infectious diseases, rare diseases, and intractable epilepsy. These categories served as variables for developing the disability risk prediction model in this study.

### Nowcasting-Based index date design for Real-Time disability risk assessment

The definition of an appropriate index date was fundamental in predictive modeling, as it served as the temporal anchor for feature extraction and outcome forecasting. An accurately specified index date ensured temporal validity, minimized information leakage, and reflected how prediction models would have been implemented in real-world clinical or administrative settings.

In this study, the index date was operationalized to simulate the point at which healthcare professionals or long-term care administrators evaluated an individual’s health status to assess disability risk. For participants who subsequently developed disability, the index date was defined as the date of disability diagnosis. For participants who remained free of disability, a random date within their observation period was assigned as the index date, conditional on the availability of at least three years of preceding health records and no prior disability events. Features were extracted from the three-year period preceding the index date (i.e., day − 1095 to day − 1) for all individuals. This approach ensured consistency in feature construction across cases and non-cases, captured temporally appropriate disease history, and maintained methodological rigor. A schematic overview of the feature construction process and index date definition was provided in Supplementary Material Figure S1.

This study adopted a nowcasting-based design, in which the model was tasked with estimating whether an individual was currently experiencing, or imminently approaching, disability status as of the index date, using only information available prior to that date. This is distinct from traditional long-term prognostic models, which aim to predict outcomes occurring within a future time frame (e.g., 6 or 12 months ahead). In contrast, nowcasting focuses on real-time risk detection and actionable prediction, making it particularly suitable for policy applications that require high-frequency surveillance and rapid identification of high-risk groups.

Our modeling strategy was designed to support batch prediction on any given calendar day, using only pre-index historical data, while avoiding any post-index leakage. In practical deployment, the model can be used to generate updated risk scores and high-risk lists at regular intervals, enabling timely outreach, functional assessment, or care resource reallocation by government agencies.

### Feature selection

This study adopted an embedded feature selection method to refine the accuracy of machine learning models in handling high-dimensional datasets [33–35]. Tree-based machine learning algorithms calculated the Gini or entropy value of each feature, evaluating its informational contribution. Features were then ranked based on the accumulated Gini or entropy values, with a threshold set at 1.25 times the average. Features that surpassed this threshold were retained as predictors, while those falling below were excluded, ensuring that only features with significant predictive power were utilized.

In this study, tree-based algorithms (Decision Tree, Random Forest, XGBoost, and LightGBM) were utilized for feature selection, creating a hierarchy of features based on their weights. To gauge the efficacy of these feature selection models, five distinct machine learning algorithms were applied to the chosen predictor sets. The predictive performance of these sets was assessed by calculating the AUC after executing each algorithm ten times. The features identified by the embedded methods were detailed in Supplementary Material Table S3. The analysis revealed that the feature set selected by LightGBM, comprising 36 features, Surpassed the other sets in performance. Although a comprehensive feature set containing all 125 predictors achieved the highest AUC, the LightGBM feature set (FSM-LightGBM) demonstrated comparable AUC efficacy with only 36 predictors.

**Fig. 2:**
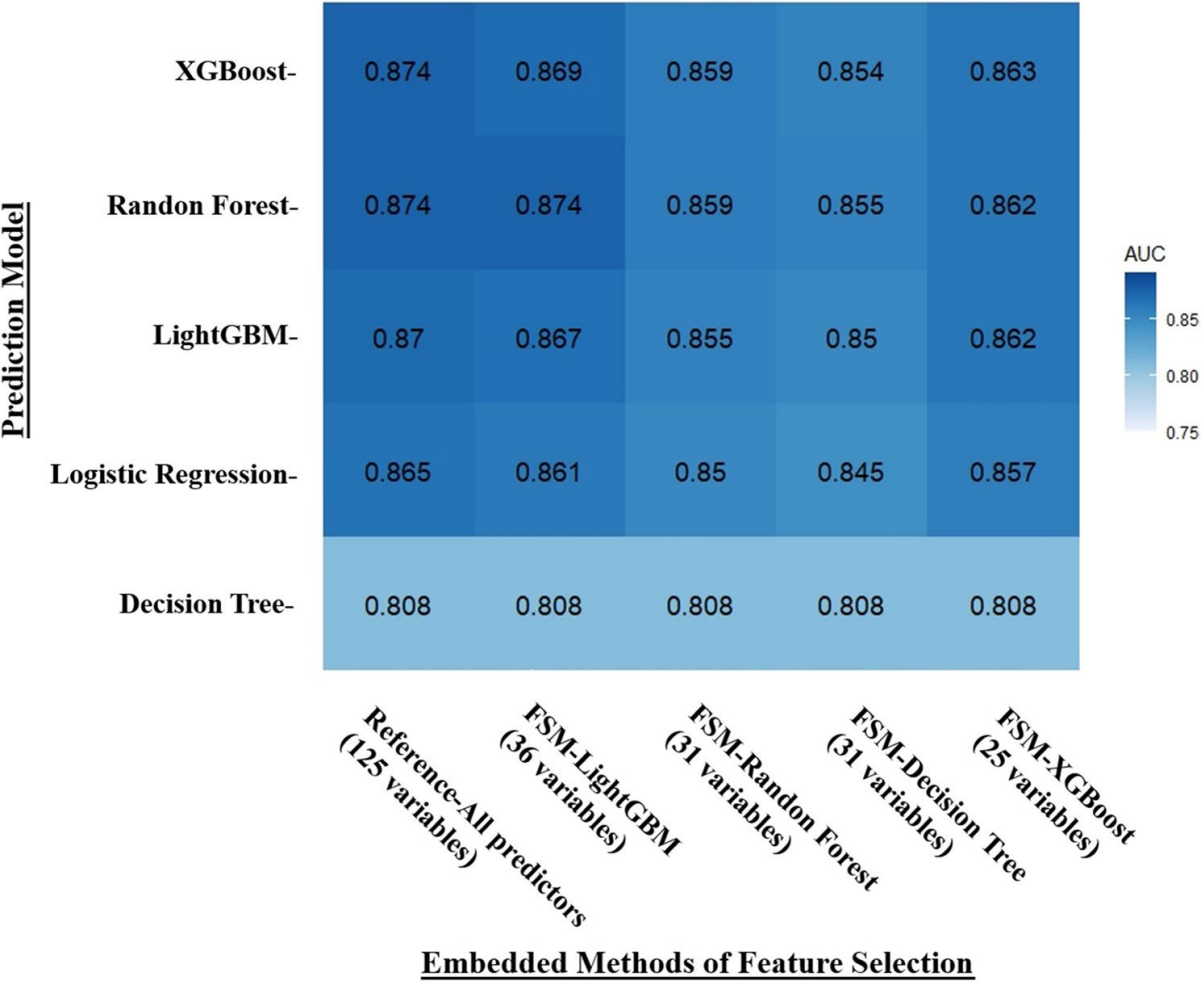
Heatmap of the AUC of selected-feature sets selected by embedded feature selection models. The y-axis represents the machine learning models (XGBoost, Random Forest, LightGBM, Logistic Regression, Decision Tree) utilized to validate the predictive performance of feature sets selected by various feature selection approaches. The x-axis represents the embedded feature selection approaches that use four tree-based machine learning models, namely XGBoost, Random Forest, LightGBM, Regression, and Decision Tree, for feature selection. The number of features selected by each embedded model is displayed. The values within each heatmap cell represent the AUC obtained by each machine learning predictive model using the corresponding selected feature set. The AUC values were calculated based on the training sets. For example, the AUC obtained by LightGBM using the FSM-Random Forest feature set (31 variables) is 0.855. And darker blue color indicates higher AUC

However, this study considered not only the number of selected features but also the overall predictive performance and stability across different machine learning models. As illustrated in Fig. 2, while FSM-XGBoost (25 variables) achieved similar AUCs, FSM-LightGBM (36 variables) demonstrated slightly higher and more stable performance across models including XGBoost, Random Forest, LightGBM, and Logistic Regression. Given our aim to construct a robust and generalizable disability prediction model, we selected the FSM-LightGBM feature set for further development, as illustrated in the heatmap presented in Fig. 2.

### Machine learning algorithms

This study employed multiple supervised machine learning (ML) algorithms, including Logistic Regression, Decision Tree, XGBoost, Random Forest, and LightGBM, to classify and predict disability among elderly adults. These ML algorithms generated interpretable predictors that facilitated the understanding of the decision-making process, resulting in ML models [36, 37]. The interpretable predictors selected by the ML algorithms could be leveraged to develop prevention strategies for disability. All ML models in this study were trained and validated using R version 4.3.0.

### Dealing with imbalanced data

The study yielded a disability outcome rate of approximately 10%, indicating an imbalanced data scenario that could diminish the effectiveness of the disability risk prediction model in classification and prediction tasks. The literature suggested the use of oversampling and undersampling techniques to address such data imbalances [38–40]. Nevertheless, empirical evidence indicated that in cases of high-dimensional data with ample samples, undersampling could outperform oversampling in terms of predictive accuracy [41, 42].

Given the magnitude of the dataset in this study, with 433,623 minority class instances and 125 predictive variables, a random undersampling approach was utilized. This technique involved randomly selecting a Subset from the group of elderly adults without disabilities to match the size of the group with disabilities, thereby balancing the ratio of individuals with and without disabilities and enhancing the predictive capacity without risking overfitting. Moreover, a 1:1 ratio was employed for the undersampling to maximize the model’s predictive performance.

### Model validation

To evaluate the performance of the disability risk prediction model, this study adopted the 5-fold cross-validation approach. This method randomly divided the data into five equal folds, with four folds used for model training and one fold used for validation. This process was repeated five times so that each fold was used once as the validation set. The resulting performance metrics were averaged to assess the model’s robustness and its generalization ability to new data, thereby preventing overfitting and providing accurate performance estimates [43].

## Results

### Predictive ability and performance validation

The predictive performance metrics (accuracy, sensitivity, specificity, balanced accuracy, and AUC) were calculated for each fold in the 5-fold cross-validation, and the mean value for each metric across the five folds was reported in Table 2 and 3. Five machine learning models were constructed using the FSM-LightGBM selected feature set (36 features). Based on the results presented in Table 2, the performance of each model in the training sets was similar to that in the testing sets, indicating that the prediction models did not suffer from overfitting.

In the testing sets, LightGBM achieved the highest sensitivity (0.8379), Random Forest achieved the highest balanced accuracy (0.7917), and XGBoost achieved the highest AUC (0.8674). Among these predictive models, XGBoost demonstrated the best average performance. The performance of XGBoost using the FSM-LightGBM feature set was similar to that using the reference feature set. This suggests that a model with fewer features performs about as good as a model with all features. Based on overall predictive performance and model stability, we selected the XGBoost model trained with the FSM-LightGBM feature set as the final model for subsequent analysis.

In addition, predictive models constructed using other feature sets—including FSM-Random Forest, FSM-Decision Tree, and FSM-XGBoost—were also evaluated, and their respective performance metrics were presented in Supplementary Material Table S4.

**Table 2:** The performance metrics of each predictive model based on 5-fold cross-validation using reference and FSM-LightGBM feature-selected sets within the Training sets

Feature-selected model	Predictive Model	Accuracy	Sensitivity	Specificity	Balanced Accuracy	AUC
Reference features set (All predictors – 125 features)	Random Forest	0.7661	0.8362	0.7575	0.7968	0.8745
	XGBoost	0.7695	0.8382	0.7610	0.7996	0.8738
	Logistic Regression	0.7975	0.7751	0.8003	0.7877	0.8651
	Decision Tree	0.7538	0.7441	0.7550	0.7496	0.8082
	LightGBM	0.7631	0.8407	0.7536	0.7972	0.8704
FSM-LightGBM (36 features)	Random Forest	0.7617	0.8401	0.7520	0.7960	0.8740
	XGBoost	0.7652	0.8345	0.7566	0.7955	0.8692
	Logistic Regression	0.7961	0.7690	0.7995	0.7842	0.8611
	Decision Tree	0.7544	0.7428	0.7559	0.7493	0.8084
	LightGBM	0.7598	0.8384	0.7501	0.7943	0.8667

**Table 3:** The performance metrics of each predictive model based on 5-fold cross-validation using reference and FSM-LightGBM feature-selected sets within the Test sets

Feature-selected model	Predictive Model	Accuracy	Sensitivity	Specificity	Balanced Accuracy	AUC
Reference features set (All predictors – 125 features)	Random Forest	0.7635	0.8291	0.7554	0.7923	0.8650
	XGBoost	0.7689	0.8355	0.7606	0.7981	0.8716
	Logistic Regression	0.7975	0.7751	0.8002	0.7877	0.8651
	Decision Tree	0.7538	0.7441	0.7550	0.7496	0.8082
	LightGBM	0.7631	0.8403	0.7536	0.7969	0.8700
FSM-LightGBM (36 features)	Random Forest	0.7588	0.8337	0.7496	0.7917	0.8634
	XGBoost	0.7648	0.8326	0.7564	0.7945	0.8674
	Logistic Regression	0.7961	0.7689	0.7995	0.7842	0.8611
	Decision Tree	0.7544	0.7427	0.7559	0.7493	0.8082
	LightGBM	0.7597	0.8379	0.7500	0.7940	0.8665

### Feature importance ranked via predictive models

This study utilized the Shapley Additive Explanation (SHAP) method to generate post-hoc explanations for the machine learning model and to provide additional information for the development of disability prevention policies [44–47]. SHAP was used to assess the impact and directionality of disability-related features. Shapley values quantified the contribution of each feature to the model’s prediction for a given instance based on cooperative game theory. A higher absolute SHAP value indicated a greater impact of the feature on the prediction outcome. In addition, the sign of the SHAP value indicated the direction of the feature’s effect: a positive SHAP value suggested that the presence or increase of a feature contributed to a higher predicted risk of disability, whereas a negative SHAP value suggested that the presence or increase of a feature contributed to a lower predicted risk. For example, a positive SHAP value for hypertension indicated that having hypertension increased the predicted risk of disability, while a negative SHAP value for younger age indicated that being younger decreased the predicted risk.

**Fig. 3:**
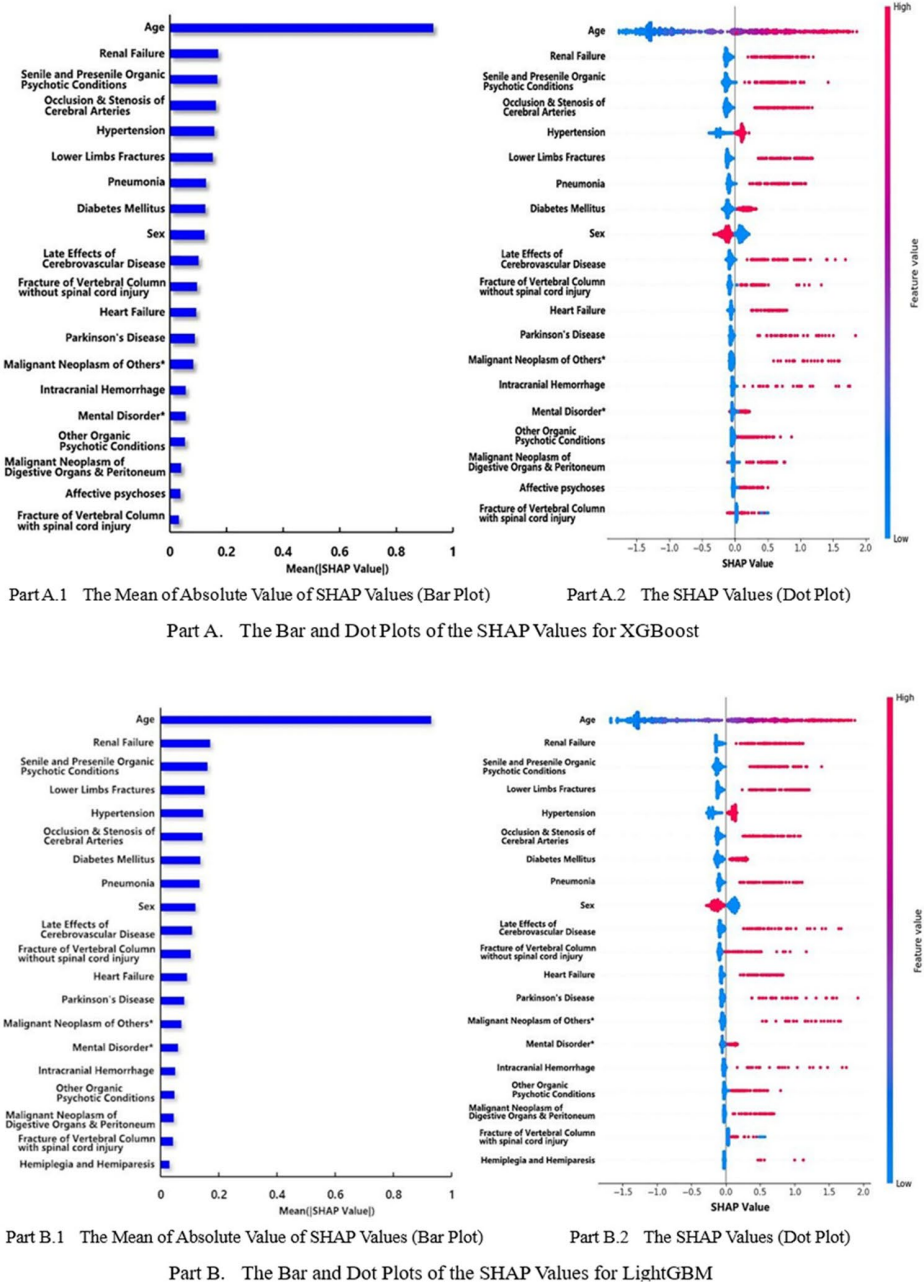
Shapley value plots of the top 20 important features of XGBoost and lightGBM. * Malignant neoplasm of others include eye, brain, thyroid gland, unspecified parts of nervous system, other endocrine glands, secondary malignant neoplasm. † Mental disorders include neurotic disorders, personality disorders, other psychotic mental disorder

The feature importance rankings of XGBoost, calculated by averaging the absolute values of Shapley values, were displayed in the bar chart in part A.1 of Fig. 3. The top 10 features with the greatest rankings were age, renal failure, dementia, cerebral vascular occlusion and stenosis, hypertension, lower extremity fractures, pneumonia, diabetes, sex, and late effects of cerebrovascular disease. The robustness of these results was confirmed by the high similarity observed between the feature importance rankings of XGBoost and LightGBM.

Part A.2 of Fig. 3 illustrated the SHAP values of XGBoost in a dot plot, where each dot represented an individual observation in the dataset, and its color indicated the observed feature value. Binary features were displayed strictly in blue or red, with blue indicating the absence of a condition and red indicating its presence. Continuous features ranged from low values (blue) to high values (red). Additionally, features with SHAP values greater than zero were associated with an increased risk of disability among elderly adults, whereas features with SHAP values less than zero were associated with a decreased risk of disability among elderly adults.

The analysis of comorbidities revealed that the majority of blue dots with negative Shapley values indicated that individuals without comorbidities had a lower risk of disability, whereas predominantly red dots with positive Shapley values indicated that individuals with comorbidities were at a higher risk of disability. Additionally, younger individuals (blue) with negative Shapley values had a lower risk of disability. Finally, women (blue) were shown to have a higher risk of disability than men (red).

## Discussion

Elderly adults with disabilities imposed a significant financial burden on medical and care systems, making the planning of long-term care resources an important public health issue [2, 5, 6]. Therefore, this study established a disease-based disability risk prediction model. The results showed that the predictive model developed using the XGBoost algorithm exhibited the best predictive performance, providing real-time disability prediction information that could be used for developing long-term care resource allocation policies. Furthermore, the XGBoost prediction model identified several key diseases associated with an increased risk of disability based on Shapley values, including chronic conditions such as renal failure, dementia, cerebral infarction and stenosis, hypertension, lower limb fractures, pneumonia, and diabetes, all of which have been shown to be highly correlated with disability [48–55]. Prevention and treatment strategies targeted at these chronic diseases may effectively reduce the burden and risk of disability in the aging population.

Current literature emphasized that the most critical step in formulating strategies for the prevention and treatment of disability in elderly adults, as well as mechanisms for allocating care resources, was to identify groups at high risk of disability [2, 5, 7, 13]. For example, Kingston et al. (2018) utilized a microsimulation model, incorporating multiple diseases as predictive parameters, to estimate the number of elderly adults with severe disabilities in the UK over the next 20 years, thereby simulating future care needs [2]. However, most existing studies on disability risk prediction for elderly adults primarily relied on data obtained from questionnaire surveys [6, 14–17]. Although such surveys captured detailed information on respondents’ physical activity, disease status, psychological conditions, and social interactions, they required substantial manpower, time, and financial resources. Moreover, the long survey cycles made it difficult to rapidly and accurately reflect the scale and distribution of elderly adults with disabilities at specific time points.

To overcome these limitations, this study utilized a national health insurance database, enabling real-time tracking of patients’ disease statuses at various time points, and incorporated a machine learning model to predict disability risk. By leveraging continuously updated dynamic disease information, the model promptly identified elderly individuals at high risk of disability and rapidly provided real-time estimates of the number and geographic distribution of elderly adults with disabilities, thereby offering timely and valuable references for governmental health agencies in planning long-term care resource allocation policies.

Furthermore, the predictive model in this study effectively utilized longitudinal health insurance data from aging populations at the national or regional level to estimate the likelihood of future disability for each individual. Based on the predicted probabilities, the model categorized individuals into low-, medium-, and high-risk groups. Government agencies could develop targeted early intervention policies tailored to the disease characteristics and specific needs of high-risk groups, thereby preventing or delaying the progression of disability and enhancing the precision and efficiency of care resource allocation.

The observed improvement in predictive accuracy when employing diseases as key predictive factors in disability models likely originated from the frequent correlation between various diseases and disabilities [10, 12, 48–55]. This association was particularly pronounced in elderly adults, whose disabilities often correlated with age-related diseases such as hypertension, diabetes, neovascular disorders, joint diseases, and respiratory ailments [9, 28]. Much of the existing research has explored the relationships between specific diseases and disabilities. Conditions such as kidney failure [52, 53], pneumonia [51], lower limb fractures [54], and diabetes [48] were recognized contributors to physical weakness and reduced muscle strength, leading to deterioration in physical capabilities and eventual disability. Similarly, cognitive deficits resulting in disability were frequently linked to dementia [50], cerebral infarction or stenosis [55], and hypertension [49].

Further studies indicated that elderly adults with multiple diseases were more prone to physical functional decline, with common multimorbidity combinations involving hypertension, cardiovascular and lung diseases, diabetes, cancer, arthritis, stroke, cognitive impairments, or severe depressive symptoms [12, 27, 29]. Kingston et al. (2018) projected that the number of elderly adults with four or more diseases would double between 2015 and 2035 [13], potentially driving an increase in disabilities among the elderly population.

The final predictive model was developed using XGBoost with the FSM-LightGBM feature set, ensuring the consideration of elderly adults with multiple concurrent diseases. The disease-disability correlations estimated by the model aligned with existing literature [8, 12, 24, 27, 28, 48, 51, 52]. Unlike previous studies, the model assigned a ranking of disease importance, identifying key risk factors for disability. According to the model, diseases were prioritized based on SHAP value rankings, listed from the most to the least important: renal failure, dementia, cerebral infarction or stenosis, hypertension, lower limb fractures, pneumonia, and diabetes. This ranking could aid in focusing prevention and treatment efforts on the most impactful diseases, thus enhancing resource allocation for effective disability management.

The disability risk prediction model constructed using the XGBoost algorithm with a feature set selected by the FSM-LightGBM method and based on a national medical claim database contrasted with traditional survey-based disability risk prediction models [6, 14, 15, 17]. It enabled real-time, cost-effective, and periodic assessment of elderly disability nationwide, overcoming the limitations of traditional surveys in terms of respondent numbers, duration, and cost. Hence, this approach was more adept at swiftly identifying groups of elderly adults in Taiwan who were at high risk for disability.

This study had several limitations. First, it primarily focused on disease variables, giving less consideration to socioeconomic factors, physical activity, and social capabilities. However, the predictive performance of the model still surpassed that of previous models incorporating these variables. Second, the evaluation of disease was based on diagnosis confirmation rather than disease severity, due to dataset constraints. Visit frequency might not have fully captured the severity or progression of chronic conditions. Third, although a three-visit confirmation criterion was applied to enhance diagnostic accuracy and minimize misclassification bias, stable chronic conditions with fewer medical visits might have been slightly underestimated. Additionally, due to the lack of detailed information on healthcare accessibility, healthcare-seeking behaviors, and familial or community support in the claims data, residual confounding could not be entirely addressed.

## Conclusions

This study developed a disease-based disability risk prediction model using nationwide health insurance claims data. The model provided timely information to identify elderly individuals at high risk of disability and to estimate the prevalence of disability across the population. By capturing disease patterns associated with disability, it assisted in decision-making for disability prevention and medical resource planning.

The model served as a supportive tool within routine administrative workflows by incorporating claims-based risk assessments, thereby assisting policymakers and healthcare administrators in identifying priority populations and informing the allocation of long-term care services. Its application contributed to more targeted planning and more efficient utilization of limited care resources.

## Supplementary Information

Supplementary Material 1.

## Data Availability

The data supporting the findings of this study are available from the Department of Long-Term Care, Ministry of Health and Welfare, Taiwan. However, restrictions apply to the availability of these data, which were used under license for the current study, and therefore are not publicly available. Data are available from the authors upon reasonable request and with permission of Department of Long-Term Care, Ministry of Health and Welfare, Taiwan.

## Abbreviations

NHIRD: National Health Insurance Research Database
LTCD: Long-Term Care Database
SHAP: Shapley Additive Explanation
CMS: Long-term care Case-Mix System
ADL: Activities of daily living
IADL: Instrumental Activities of Daily Living
AUC: Area under the receiver operating characteristic curve
ML: Machine learning
FSM-LightGBM: Feature Selection Model using Light Gradient Boosting Machine (assumed context based on the description)

## References

[1] Prince MJ, Wu F, Guo Y, Gutierrez Robledo LM, O’Donnell M, Sullivan R, et al. The burden of disease in older people and implications for health policy and practice. Lancet. 2015;385(9967):549–62. 25468153 10.1016/S0140-6736(14)61347-7

[2] Kingston A, Comas-Herrera A, Jagger C. Forecasting the care needs of the older population in England over the next 20 years: estimates from the population ageing and care simulation (PACSim) modelling study. Lancet Public Health. 2018;3(9):e447-55. 30174210 10.1016/S2468-2667(18)30118-X PMC6123499

[3] Bloom DE, Chatterji S, Kowal P, Lloyd-Sherlock P, McKee M, Rechel B, et al. Macroeconomic implications of population ageing and selected policy responses. Lancet. 2015;385(9968):649–57. 25468167 10.1016/S0140-6736(14)61464-1 PMC4469267

[4] Chang AY, Skirbekk VF, Tyrovolas S, Kassebaum NJ, Dieleman JL. Measuring population ageing: an analysis of the global burden of disease study 2017. Lancet Public Health. 2019;4(3):e159–67. 30851869 10.1016/S2468-2667(19)30019-2 PMC6472541

[5] Taş Ü, Steyerberg EW, Bierma-Zeinstra SMA, Hofman A, Koes BW, Verhagen AP. Age, gender and disability predict future disability in older people: the Rotterdam study. BMC Geriatr. 2011;11(1):22. 21569279 10.1186/1471-2318-11-22 PMC3224098

[6] Jonkman NH, Colpo M, Klenk J, Todd C, Hoekstra T, Del Panta V, et al. Development of a clinical prediction model for the onset of functional decline in people aged 65–75 years: pooled analysis of four European cohort studies. BMC Geriatr. 2019;19(1):179. 31248370 10.1186/s12877-019-1192-1 PMC6595632

[7] Winblad I, Jääskeläinen M, Kivelä S-L, Hiltunen P, Laippala P. Prevalence of disability in three birth cohorts at old age over time spans of 10 and 20 years. J Clin Epidemiol. 2001;54(10):1019–24. 11576813 10.1016/s0895-4356(01)00370-5

[8] Li C-L, Chiu Y-C, Chang H-Y, Hsu K-H, Bai Y-B, Wang H-H. Association of geriatric conditions and cardiovascular diseases with disability in older adults with diabetes: findings from a nationally representative survey. Geriatr Gerontol Int. 2013;13(3):563–70. 22985021 10.1111/j.1447-0594.2012.00935.x

[9] Klijs B, Nusselder WJ, Looman CW, Mackenbach JP. Contribution of chronic disease to the burden of disability. PLoS ONE. 2011;6(9):e25325. 21966497 10.1371/journal.pone.0025325 PMC3178640

[10] Sharma P, Maurya P, Muhammad T. Number of chronic conditions and associated functional limitations among older adults: cross-sectional findings from the longitudinal aging study in India. BMC Geriatr. 2021;21(1):664. 34814856 10.1186/s12877-021-02620-0 PMC8609791

[11] Barnett K, Mercer SW, Norbury M, Watt G, Wyke S, Guthrie B. Epidemiology of multimorbidity and implications for health care, research, and medical education: a cross-sectional study. Lancet. 2012;380(9836):37–43. 22579043 10.1016/S0140-6736(12)60240-2

[12] Ryan A, Wallace E, O’Hara P, Smith SM. Multimorbidity and functional decline in community-dwelling adults: a systematic review. Health Qual Life Outcomes. 2015;13(1):168. 26467295 10.1186/s12955-015-0355-9 PMC4606907

[13] Kingston A, Robinson L, Booth H, Knapp M, Jagger C, project ftM. Projections of multi-morbidity in the older population in England to 2035: estimates from the population ageing and care simulation (PACSim) model. Age Ageing. 2018;47(3):374–80. 29370339 10.1093/ageing/afx201 PMC5920286

[14] Xiang C, Wu Y, Jia M, Fang Y. Machine learning-based prediction of disability risk in geriatric patients with hypertension for different time intervals. Arch Gerontol Geriatr. 2023;105:104835. 36335673 10.1016/j.archger.2022.104835

[15] Yu Q, Li Z, Yang C, Zhang L, Xing M, Li W. Predicting functional dependency using machine learning among a middle-aged and older Chinese population. Arch Gerontol Geriatr. 2023;115:105124. 37454417 10.1016/j.archger.2023.105124

[16] Saarela M, Ryynänen O-P, Äyrämö S. Predicting hospital associated disability from imbalanced data using supervised learning. Artif Intell Med. 2019;95:88–95. 30292537 10.1016/j.artmed.2018.09.004

[17] Zhang L, Cui H, Chen Q, Li Y, Yang C, Yang Y. A web-based dynamic nomogram for predicting instrumental activities of daily living disability in older adults: a nationally representative survey in China. BMC Geriatr. 2021;21(1):311. 34001030 10.1186/s12877-021-02223-9 PMC8127258

[18] Lahbib H, Mandereau-Bruno L, Goria S, Wagner V, Torres MJ, Féart C, et al. Development and indirect validation of a model predicting frailty in the French healthcare claims database. Sci Rep. 2025;15(1):11344. 40175586 10.1038/s41598-025-95629-z PMC11965288

[19] Kim DH, Schneeweiss S, Glynn RJ, Lipsitz LA, Rockwood K, Avorn J. Measuring frailty in medicare data: development and validation of a Claims-Based frailty index. Journals Gerontology: Ser A. 2017;73(7):980–7. 10.1093/gerona/glx229 PMC6001883 29244057

[20] Lammi U-K, Kivelä S-L, Nissinen A, Punsar S, Puska P, Karvonen M. Predictors of disability in elderly Finnish men—A longitudinal study. J Clin Epidemiol. 1989;42(12):1215–25. 2585012 10.1016/0895-4356(89)90120-0

[21] Orfila F, Carrasco-Ribelles LA, Abellana R, Roso-Llorach A, Cegri F, Reyes C, et al. Validation of an electronic frailty index with electronic health records: eFRAGICAP index. BMC Geriatr. 2022;22(1):404. 35525922 10.1186/s12877-022-03090-8 PMC9080132

[22] Kinosian B, Wieland D, Gu X, Stallard E, Phibbs CS, Intrator O. Validation of the JEN frailty index in the National Long-Term care survey community population: identifying functionally impaired older adults from claims data. BMC Health Serv Res. 2018;18(1):908. 30497450 10.1186/s12913-018-3689-2 PMC6267903

[23] Gontijo Guerra S, Djamal B, Vasiliadis H-M. Changes in instrumental activities of daily living functioning associated with concurrent common mental disorders and physical Multimorbidity in older adults. Disabil Rehabil. 2021;43(25):3663–71. 32255362 10.1080/09638288.2020.1745303

[24] Forjaz MJ, Rodriguez-Blazquez C, Ayala A, Rodriguez-Rodriguez V, de Pedro-Cuesta J, Garcia-Gutierrez S, et al. Chronic conditions, disability, and quality of life in older adults with multimorbidity in Spain. Eur J Intern Med. 2015;26(3):176–81. 25724771 10.1016/j.ejim.2015.02.016

[25] Costa Filho AM, Mambrini JVM, Malta DC, Lima-Costa MF, Peixoto SV, National Health Survey. Contribution of chronic diseases to the prevalence of disability in basic and instrumental activities of daily living in elderly Brazilians: the (2013). Cadernos de Saúde Pública. 2018;34(1):e00204016. 10.1590/0102-311X00204016 29412330

[26] Raina P, Gilsing A, Mayhew AJ, Sohel N, van den Heuvel E, Griffith LE. Individual and population level impact of chronic conditions on functional disability in older adults. PLoS ONE. 2020;15(2):e0229160. 32078637 10.1371/journal.pone.0229160 PMC7032687

[27] Quiñones AR, Markwardt S, Botoseneanu A. Multimorbidity combinations and disability in older adults. J Gerontol A Biol Sci Med Sci. 2016;71(6):823–30. 26968451 10.1093/gerona/glw035 PMC4888400

[28] Su P, Ding H, Zhang W, Duan G, Yang Y, Chen R, et al. The association of multimorbidity and disability in a community-based sample of elderly aged 80 or older in Shanghai, China. BMC Geriatr. 2016;16(1):178. 27784269 10.1186/s12877-016-0352-9 PMC5081877

[29] Arokiasamy P, Uttamacharya n, Jain K, Multi-Morbidity. Functional limitations, and Self-Rated health among older adults in India:Cross-Sectional analysis of LASI pilot survey, 2010. SAGE Open. 2015;5(1):2158244015571640.

[30] Yu H-W, Tu Y-K, Kuo P-H, Chen Y-M. Use of home- and community-based services in taiwan’s national 10-year long-term care plan. J Appl Gerontol. 2020;39(7):722–30. 29766761 10.1177/0733464818774642

[31] Chen CF, Fu TH. Policies and transformation of long-term care system in Taiwan. Ann Geriatr Med Res. 2020;24(3):187–94. 33012140 10.4235/agmr.20.0038 PMC7533198

[32] Hsu H-C, Chen C-F. LTC 2.0: the 2017 reform of home- and community-based long-term care in Taiwan. Health Policy. 2019;123(10):912–6. 31455563 10.1016/j.healthpol.2019.08.004

[33] Liu H, Zhou M, Liu Q. An embedded feature selection method for imbalanced data classification. IEEE/CAA Journal of Automatica Sinica. 2019;6(3):703–15.

[34] Chen C-W, Tsai Y-H, Chang F-R, Lin W-C. Ensemble feature selection in medical datasets: combining filter, wrapper, and embedded feature selection results. Expert Syst. 2020;37(5):e12553.

[35] Cai J, Luo J, Wang S, Yang S. Feature selection in machine learning: a new perspective. Neurocomputing. 2018;300:70–9.

[36] Vilone G, Longo L. Notions of explainability and evaluation approaches for explainable artificial intelligence. Inf Fusion. 2021;76:89–106.

[37] Belle V, Papantonis I. Principles and practice of explainable machine learning. Front Big Data. 2021;Volume 4–2021. 10.3389/fdata.2021.688969 PMC8281957 34278297

[38] Amin A, Anwar S, Adnan A, Nawaz M, Howard N, Qadir J, et al. Comparing oversampling techniques to handle the class imbalance problem: a customer churn prediction case study. IEEE Access. 2016;4:7940–57.

[39] Liu XY, Wu J, Zhou ZH. Exploratory undersampling for class-imbalance learning. IEEE Trans Syst Man Cybernetics Part B (Cybernetics). 2009;39(2):539–50. 10.1109/TSMCB.2008.2007853 19095540

[40] Lee PH. Resampling methods improve the predictive power of modeling in class-imbalanced datasets. Int J Environ Res Public Health. 2014;11(9):9776–89. 25238271 10.3390/ijerph110909776 PMC4199049

[41] Naseriparsa M, Kashani MMR. Combination of PCA with SMOTE resampling to boost the prediction rate in lung cancer dataset. Int J Comput Appl. 2013(3). 10.5120/13376-0987.

[42] Blagus R, Lusa L. Smote for high-dimensional class-imbalanced data. BMC Bioinformatics. 2013;14(1):106. 23522326 10.1186/1471-2105-14-106 PMC3648438

[43] Jung Y. Multiple predicting K-fold cross-validation for model selection. J Nonparametr Stat. 2018;30(1):197–215.

[44] Heskes T, Sijben E, Bucur IG, Claassen T. Causal shapley values: exploiting causal knowledge to explain individual predictions of complex models. Adv Neural Inf Process Syst. 2020;33:4778–89.

[45] Aas K, Jullum M, Løland A. Explaining individual predictions when features are dependent: more accurate approximations to Shapley values. Artif Intell. 2021;298:103502.

[46] Nohara Y, Matsumoto K, Soejima H, Nakashima N. Explanation of machine learning models using Shapley additive explanation and application for real data in hospital. Comput Methods Programs Biomed. 2022;214:106584. 34942412 10.1016/j.cmpb.2021.106584

[47] Lundberg SM, Lee S-I. A unified approach to interpreting model predictions. Adv Neural Inf Process Syst. 2017;30.

[48] Park SW, Goodpaster BH, Strotmeyer ES, Kuller LH, Broudeau R, Kammerer C, et al. Accelerated loss of skeletal muscle strength in older adults with type 2 diabetes: the health, aging, and body composition study. Diabetes Care. 2007;30(6):1507–12. 17363749 10.2337/dc06-2537

[49] Di Bari M, Pahor M, Franse LV, Shorr RI, Wan JY, Ferrucci L, et al. Dementia and disability outcomes in large hypertension trials: lessons learned from the systolic hypertension in the elderly program (SHEP) trial. Am J Epidemiol. 2001;153(1):72–8. 11159149 10.1093/aje/153.1.72

[50] Sauvaget C, Yamada M, Fujiwara S, Sasaki H, Mimori Y. Dementia as a predictor of functional disability: a four-year follow-up study. Gerontology. 2002;48(4):226–33. 12053112 10.1159/000058355

[51] Park CM, Kim W, Rhim HC, Lee ES, Kim JH, Cho KH, et al. Frailty and hospitalization-associated disability after pneumonia: a prospective cohort study. BMC Geriatr. 2021;21(1):111. 33546614 10.1186/s12877-021-02049-5 PMC7864132

[52] Greco A, Paroni G, Seripa D, Addante F, Dagostino MP, Aucella F. Frailty, disability and physical exercise in the aging process and in chronic kidney disease. Kidney Blood Press Res. 2014;39(2–3):164–8. 25117919 10.1159/000355792

[53] Tamura MK, Covinsky KE, Chertow GM, Yaffe K, Landefeld CS, McCulloch CE. Functional status of elderly adults before and after initiation of dialysis. N Engl J Med. 2009;361(16):1539–47. 19828531 10.1056/NEJMoa0904655 PMC2789552

[54] den Ouden MEM, Schuurmans MJ, Arts IEMA, van der Schouw YT. Physical performance characteristics related to disability in older persons: a systematic review. Maturitas. 2011;69(3):208–19. 21596497 10.1016/j.maturitas.2011.04.008

[55] Strambo D, Bartolini B, Beaud V, Marto JP, Sirimarco G, Dunet V, et al. Thrombectomy and thrombolysis of isolated posterior cerebral artery occlusion. Stroke. 2020;51(1):254–61. 31718503 10.1161/STROKEAHA.119.026907

