# Supplementary material for: Advancing healthcare allocation and prevention of disability: the role of disease-based predictive model for disability in aging adults
Source: BMC Geriatr. 2025 Oct 21;25:792. doi: 10.1186/s12877-025-06457-9 (PMC12539223; doi:10.1186/s12877-025-06457-9)
Supplement: Supplementary file 1 — Supplementary Material 1. [file 12877_2025_6457_MOESM1_ESM.docx]

**Supplementary Material**

**S1. CMS Levels with Corresponding ADL/IADL Disability Measures and Subsidy Allocations**

**Table S1.** CMS Levels with Corresponding ADL/IADL Disability Measures and Subsidy Allocations

| **CMS Level** | **Corresponding ADL/IADL Disability** | **Subsidy Items and Amounts** |
| --- | --- | --- |
| Level 1a | No functional limitation  (Diagnosed with dementia but no ADL Disability) | No subsidy |
| Level 1b | No functional limitation  (Frail older adults with IADL Disability) | No subsidy |
| Level 2 | Mild disability  (ADL: 91–99, IADL: 21–23) | - Home-based and professional care services: NT$10,020 - Transportation services for medical or rehabilitation visits: NT$1,680–2,400 - Assistive device provision and home environment modification: NT$40,000 - Respite care services: NT$32,340 |
| Level 3 | Mild disability  (ADL: 61–90, IADL: 18–20) | - Home-based and professional care services: NT$15,460 - Transportation services for medical or rehabilitation visits: NT$1,680–2,400 - Assistive device provision and home environment modification: NT$40,000 - Respite care services: NT$32,340 |
| Level 4 | Moderate disability  (ADL: 31–60, IADL: 15–17) | - Home-based and professional care services: NT$18,580 - Transportation services for medical or rehabilitation visits: NT$1,680–2,400 - Assistive device provision and home environment modification: NT$40,000 - Respite care services: NT$32,340 |
| Level 5 | Moderate disability  (ADL: 16–30, IADL: 12–14) | - Home-based and professional care services: NT$24,100 - Transportation services for medical or rehabilitation visits: NT$1,680–2,400 - Assistive device provision and home environment modification: NT$40,000 - Respite care services: NT$32,340 |
| Level 6 | Moderate disability  (ADL: 11–15, IADL: 9–11) | - Home-based and professional care services: NT$28,070 - Transportation services for medical or rehabilitation visits: NT$1,680–2,400 - Assistive device provision and home environment modification: NT$40,000 - Respite care services: NT$32,340 |
| Level 7 | Severe disability  (ADL: 6–10, IADL: 6–8) | - Home-based and professional care services: NT$32,090 - Transportation services for medical or rehabilitation visits: NT$1,680–2,400 - Assistive device provision and home environment modification: NT$40,000 - Respite care services: NT$48,510 |
| Level 8 | Severe disability  (ADL: 0–5, IADL: 0–5) | - Home-based and professional care services: NT$36,180 - Transportation services for medical or rehabilitation visits: NT$1,680–2,400 - Assistive device provision and home environment modification: NT$40,000 - Respite care services: NT$48,510 |

Note: The “Corresponding ADL/IADL Disability” reflects only one component of the overall CMS assessment. In practice, CMS levels are determined through a comprehensive evaluation conducted by trained social workers based on multiple domains within the CMS framework. Nevertheless, ADL and IADL scores can serve as a preliminary indicator to describe the functional status of the individual.

**S2. Diseases Associated with Disability and Corresponding ICD-9-CM Codes**

Table S2. The diseases related to disability and corresponding ICD-9-CM codes

Note: The numbers in parentheses refer to ICD-9-CM codes.

| **No.** | **Major diseases**  **(ICD-9-CM)** | **Minor diseases**  **(ICD-9-CM)** |
| --- | --- | --- |
| 1 | Tuberculosis of the meninges and central nervous system (013) | 1. Tuberculosis of the meninges and central nervous system (013) |
| 2 | Bone and Joint Tuberculosis (015) | 1. Bone and joint tuberculosis (015) |
| 3 | Other tuberculosis (010) | 1. Primary tuberculosis infection (010) |
| 4 | Human immunodeficiency virus infection (042) | 1. Human immunodeficiency virus infection (042) |
| 5 | Acute poliomyelitis (045) | 1. Acute poliomyelitis (045) |
| 6 | Chronic Viral Infection of the Central Nervous System (046) | 1. Chronic viral infection of the central nervous system (046) |
| 7 | Meningitis due to enterovirus (047) | 1. Meningitis due to enterovirus (047) |
| 8 | Diseases of the central nervous system caused by other enteroviruses and non-arthropod vector viruses  (048,049) | 1. Other enterovirus-induced diseases of the central nervous system (048) |
|  |  | 2. Central nervous system diseases caused by other non-arthropod vectors (049) |
| 9 | Malignant neoplasm of the lip, mouth and pharynx  (140,141,142,143,144,145,  146,147,148,149) | 1. Lip Malignant neoplasm (140) |
|  |  | 2. Malignant neoplasm of the tongue (141) |
|  |  | 3. Major salivary gland Malignant neoplasm (142) |
|  |  | 4. Malignant neoplasm of gingiva (143) |
|  |  | 5. Malignant neoplasm of the floor of the mouth (144) |
|  |  | 6. Malignant neoplasm of other parts of the mouth (145) |
|  |  | 7. Malignant neoplasm of the oropharynx (146) |
|  |  | 8. Nasopharyngeal malignancy (147) |
|  |  | 9. Hypopharyngeal malignancy (148) |
|  |  | 10. Malignant neoplasm of the lip, mouth, pharynx, and other areas of unknown demarcation (149) |
| 10 | Malignant neoplasm of digestive organs and peritoneum  (150,151,152,153,154,155  ,156,157,158,159) | 1. Esophageal Malignant neoplasm (150) |
|  |  | 2. Malignant neoplasm of the stomach (151) |
|  |  | 3. Malignant neoplasm of small intestine (including duodenum) (152) |
|  |  | 4. Malignant neoplasm of the colon (153) |
|  |  | 5. Malignant neoplasm of the rectum, rectosigmoid junction and anus (154) |
|  |  | 6. Malignant neoplasm of the liver and intrahepatic bile ducts (155) |
|  |  | 7. Malignant neoplasm of gallbladder and extrahepatic bile duct (156) |
|  |  | 8. Pancreatic malignancy (157) |
|  |  | 9. Malignant neoplasm of the posterior abdominal cavity and peritoneum (158) |
|  |  | 10. Malignant neoplasm of the digestive organs, other intraperitoneal and unspecified sites (159) |
| 11 | Malignant neoplasm of the respiratory tract and internal thoracic organs  (160,161,162,163,164,165) | 1. Malignant neoplasm of the nasal cavity, middle ear and paranasal sinuses (160) |
|  |  | 2. Malignant laryngeal tumor (161) |
|  |  | 3. Malignant neoplasm of trachea, bronchus and lung (162) |
|  |  | 4. Thoracic (rib) Malignant neoplasm (163) |
|  |  | 5. Malignant neoplasm of thymus, heart and mediastinum (164) |
|  |  | 6. Malignant neoplasm of the respiratory system, other intrathoracic organs, and unspecified sites (165) |
| 12 | Malignant neoplasm of bone, connective tissue, cortex breast  (170,171,172,173,174,175,176) | 1. Malignant neoplasm of bone and joint cartilage (170) |
|  |  | 2. Malignant neoplasm of connective tissue and other soft tissues (171) |
|  |  | 3. Skin malignant melanoma (172) |
|  |  | 4. Other Malignant neoplasm of the skin (173) |
|  |  | 5. Malignant neoplasm of female breast (174) |
|  |  | 6. Malignant neoplasm of the male breast (175) |
|  |  | 7. Kaposi's sarcoma (176) |
| 13 | Malignant neoplasm of genitourinary organs  (179,180,181,182,183,184,  185,186,187,188,189) | 1. Malignant neoplasm of uterus (179) |
|  |  | 2. Cervical Malignant neoplasm (180) |
|  |  | 3. Placental malignancy (181) |
|  |  | 4. Malignant neoplasm of the uterine body (182) |
|  |  | 5. Malignant neoplasm of ovary and other uterine appendages (183) |
|  |  | 6. Malignant neoplasm of other female genital organs (184) |
|  |  | 7. Malignant neoplasm of the prostate gland (185) |
|  |  | 8. Malignant neoplasm of testis (186) |
|  |  | 9. Malignant neoplasm of the penis and other male genital organs (187) |
|  |  | 10. Malignant neoplasm of the bladder (188) |
|  |  | 11. Malignant neoplasm of the kidney and other urinary organs (189) |
| 14 | Other malignant neoplasm and unspecified sites  (190,191,192,193,194,195,  196,197,198,199) | 1. Malignant neoplasm of the eye (190) |
|  |  | Malignant neoplasm of the brain (191) |
|  |  | 3. Malignant neoplasm of other parts of the nervous system (192) |
|  |  | 4. Malignant thyroid tumor (193) |
|  |  | 5. Malignant neoplasm of other endocrine glands and related tissues (194) |
|  |  | 6. Other Malignant neoplasm with unknown demarcation (195) |
|  |  | 7. Subsequent and unspecified Malignant neoplasm of the lymph glands (196) |
|  |  | 8. Respiratory and digestive system sequelae malignancies (197) |
|  |  | 9. Other site-specific sequelae of Malignant neoplasm (198) |
|  |  | 10. Non-specific Malignant neoplasm (199) |
| 15 | Malignant neoplasm of lymphatic and hematopoietic tissues  (200,201,202,203,204,205,  206,207,208) | 1. Lymphosarcoma and reticulocytic sarcoma (200) |
|  |  | 2. Hodgkin's (Hodgkin's) disease (201) |
|  |  | 3. Other Malignant neoplasm of lymphatic and histiocytic tissues (202) |
|  |  | 4. Myeloma multiforme and immunoproliferative tumors (203) |
|  |  | 5. Lymphatic leukemia (204) |
|  |  | 6. Myelogenous leukemia (205) |
|  |  | 7. Monoclonal leukemia (206) |
|  |  | 8. Other specific leukemia (207) |
|  |  | 9. Leukemia of unknown cell type (208) |
| 16 | Carcinoma in situ  (230,231,232,233,234) | 1. Carcinoma in situ of digestive organs (230) |
|  |  | 2. Carcinoma in situ of respiratory system(231) |
|  |  | 3. Skin carcinoma in situ (232) |
|  |  | 4. Carcinoma in situ of the breast and genitourinary tract (233) |
|  |  | 5. Carcinoma in situ of other sites (234) |
| 17 | Unspecified tumor  (235,236,237,238,239) | 1. Tumor of unspecified gastrointestinal and respiratory system (235) |
|  |  | 2. Tumor of unspecified genitourinary organs (236) |
|  |  | Endocrine gland and neurological tumor of unknown nature (237) |
|  |  | 4. Tumors of unknown nature in other sites and tissues (238) |
|  |  | 5. Tumor of unspecified nature (239) |
| 18 | Diabetes mellitus (250) | 1. Diabetes mellitus (250) |
| 19 | Other diseases of blood and blood-forming organs  (286,287,288,289) | 1. Blood clotting defects (286) |
|  |  | 2. Purpura and other hemorrhagic pathologies (287) |
|  |  | 3. White blood cell disease (288) |
|  |  | 4. Other blood and blood-forming organ diseases (289) |
| 20 | Senile and presenile organic psychotic conditions  (290) | 1. Senile and presenile organic psychotic conditions (290) |
| 21 | Alcoholic drug-related psychosis  (291,292) | 1. Alcoholic psychosis (291) |
|  |  | 2. Drug-related psychosis (292) |
| 22 | Other organic psychotic conditions (chronic)  (294) | 1. Other organic psychotic conditions (chronic) (294) |
| 23 | Emotional psychosis  (mania, depression, bipolar disorder) (296) | 1. Affective psychosis (296) |
| 24 | Other psychiatric disorders  (295,297,298,299) | 1. Schizophrenic disorders (295) |
|  |  | 2. Delusional state (297) |
|  |  | 3. Other non-organic psychiatric disorders (298) |
|  |  | 4. Childhood psychiatric disorders (299) |
| 25 | Psychotic disorders, personality disorders and other non-psychotic mental disorders  (300,301,302,303,304,305,306,  307,308,309,310,311,312,313,  314,315,316) | 1. Psychosis (300) |
|  |  | 2. Personality abnormalities (301) |
|  |  | 3. Psychosexual deviations and disorders (302) |
|  |  | 4. Alcohol addiction syndrome (303) |
|  |  | 5. Drug addiction (304) |
|  |  | 6. Drug Abuse (305) |
|  |  | 7. Psychogenic physiological dysfunction (306) |
|  |  | 8. Specific symptoms or syndromes, NEC (307) |
|  |  | 9. Acute psychological stress response (308) |
|  |  | 10. Environmental adaptation barriers (309) |
|  |  | 11. Specific non-psychiatric psychiatric disorders after organic brain injury (310) |
|  |  | 12. Depressive disorders, NEC (311) |
|  |  | 13. Behavior barriers, NEC (312) |
|  |  | 14. Mood disorders specific to childhood and adolescence (313) |
|  |  | 15. Childhood hyperactivity syndrome (314) |
|  |  | 16. Specific development delays (315) |
|  |  | 17. Psychiatric factors related to other specific diseases (316) |
| 26 | Insufficient intelligence  (317,318,319) | 1. Mild intelligence deficiency (317) |
|  |  | 2. Other specific intelligence deficiencies (318) |
|  |  | 3. Insufficient intelligence (319) |
| 27 | Encephalitis, myelitis and encephalomyelitis  (323) | 1. Encephalitis, myelitis and encephalomyelitis (323) |
| 28 | Other inflammatory diseases of the central nervous system  (321,324,325) | 1. Meningitis caused by other pathogens (321) |
|  |  | 2. Intracranial and spinal canal abscess (324) |
|  |  | 3. Intracranial venous sinus phlebitis and thrombophlebitis (325) |
| 29 | Intracranial abscess or septic infection  Subsequent Impacts  (326) | 1. Post-cranial abscess or septic infection (326) |
| 30 | Brain degeneration that often appears in childhood  (330) | 1. Brain degeneration that often appears in childhood (330) |
| 31 | Alzheimer's disease  (3310) | 1. Alzheimer's disease (3310) |
| 32 | Burkitt's disease  (3311) | 1. Bicker's disease (3311) |
| 33 | Age-related brain degeneration  (3312) | 1. Age-related brain degeneration (3312) |
| 34 | Other brain degeneration  (3313,3314,3317,3318,3319) | 1. Traffic hydrocephalus (3313) |
|  |  | 2. Obstructive hydronephrosis (3314) |
|  |  | 3. Other specific diseases caused by brain degeneration (3317) |
|  |  | 4. Other brain degeneration (3318) |
|  |  | 5. Brain degeneration (3319) |
| 35 | Parkinson's disease  (332) | 1. Parkinson's disease (332) |
| 36 | Other extracorporeal diseases and  Abnormal Action Disorders  (333) | 1. Other extracorporeal diseases and abnormal movement disorders (333) |
| 37 | Spinal microcephaly  (334) | 1. Spinal microcephaly (334) |
| 38 | Werdnig-Hoffmann's disease  (3350) | 1. Werdnig-Hoffmann's disease (3350) |
| 39 | Spinal muscular atrophy  (33510,33511,33519) | 1. Spinal muscular atrophy (33510) |
|  |  | 2. Kugelberg-Welander's disease (33511) |
|  |  | 3. Other spinal muscular atrophy (33519) |
| 40 | Motor neuron disease  (33520,33521,33522,  33523,33524,33529) | 1. Amyotrophic lateral sclerosis (33520) |
|  |  | 2. Progressive myasthenia gravis (33521) |
|  |  | 3. Progressive medullary paralysis (33522) |
|  |  | 4. Pseudomyelitis (33523) |
|  |  | 5. Primary lateral sclerosis (33524) |
|  |  | 6. Other motor neuron diseases (33529) |
| 41 | Other anterior horn cell diseases  (3358,3359) | 1. Other anterior horn cell diseases (3358) |
|  |  | 2. Anterior horn cell disease (3359) |
| 42 | Other diseases of the spinal cord  (336) | 1. Other diseases of the spinal cord (336) |
| 43 | Autonomic nervous system disorders  (337) | 1. Autonomic nervous system disorders (337) |
| 44 | Multiple Sclerosis  (340) | 1. Multiple sclerosis (340) |
| 45 | Other demyelinating diseases of the central nervous system  (341) | 1. Other demyelinating diseases of the central nervous system (341) |
| 46 | Hemiplegia and hemiparesis  (342) | 1. Hemiplegia and hemiparesis (342) |
| 47 | Infantile cerebral palsy  (343) | 1. Infantile cerebral palsy (343) |
| 48 | Other paralytic syndromes  (344) | 1. Other paralytic syndromes (344) |
| 49 | Myasthenia gravis  (3580) | 1. Myasthenia gravis (3580) |
| 50 | Other muscle nerve disorders  (3581,3582,3588,3589) | 1. Myasthenia gravis syndrome due to other specific diseases (3581) |
|  |  | 2. Toxic myoneural disorders (3582) |
|  |  | 3. Other specific myoneural disorders (3588) |
|  |  | 4. Myoneural disorders (3589) |
| 51 | Undesirable degeneration of muscle nutrition(myogenic myopathy) and other myopathies  (359) | 1. Myotonic dysplasia (myogenic myopathy) and other myopathic changes (359) |
| 52 | Other peripheral nervous system disorders  (350,351,352,353,354,  355,356,357) | 1. Trigeminal nerve disorder (350) |
|  |  | 2. Facial nerve disorders (351) |
|  |  | 3. Other neurological disorders (352) |
|  |  | 4. Nerve root and plexus disorders (353) |
|  |  | 5. Single neuritis and multiple single neuritis in the upper extremities (354) |
|  |  | 6. Single neuritis of the lower limbs (355) |
|  |  | 7. Hereditary and idiopathic peripheral neuropathy (356) |
|  |  | 8. Inflammatory and toxic neuropathy (357) |
| 53 | Other Diseases of the Retina  (362) | 1. Other disorders of the retina (362) |
| 54 | Glaucoma  (365) | 1. Glaucoma (365) |
| 55 | Blindness and low vision  (369) | 1. Blindness and low vision (369) |
| 56 | Deafness (Loss of hearing) (389) | 1. Deafness(Loss of hearing) (389) |
| 57 | Chronic rheumatic heart disease  (393,394,395,396,397,398) | 1. Chronic rheumatic pericarditis (393) |
|  |  | 2. Rheumatic mitral valve disease (394) |
|  |  | 3. Rheumatic aortic valve disease (395) |
|  |  | 4. Mitral valve and aortic valve disease (396) |
|  |  | 5. Other endocardial structural diseases (397) |
|  |  | 6. Other rheumatic heart diseases (398) |
| 58 | Hypertension disease  (401,402,403,404,405) | 1. Intrinsic hypertension (401) |
|  |  | 2. Hypertensive heart disease (402) |
|  |  | 3. Hypertensive renal disease (403) |
|  |  | 4. Hypertensive heart and kidney disease (404) |
|  |  | 5. Recurrent hypertension (405) |
| 59 | Myocardial infarction  (410,411,412) | 1. Acute myocardial infarction (410) |
|  |  | 2. Other acute and subacute ischemic heart disease (411) |
|  |  | 3. Old myocardial infarction (412) |
| 60 | Other forms of chronic ischemic heart disease  (414) | 1. Other forms of chronic ischemic heart disease (414) |
| 61 | Pulmonary circulatory heart disease  (415,416,417) | 1. Acute pulmonary heart disease (415) |
|  |  | 2. Chronic pulmonary heart disease (416) |
|  |  | 3. Other pulmonary circulatory diseases (417) |
| 62 | Cardiomyopathy  (425) | 1. Cardiomyopathy (425) |
| 63 | Heart Failure  (428) | 1. Heart failure (428) |
| 64 | Intracranial hemorrhage  (430,431,432) | 1. Subsarcoid hemorrhage (430) |
|  |  | 2. Intracerebral hemorrhage (431) |
|  |  | 3. Other intracranial hemorrhage (432) |
| 65 | Occlusion and stenosis of cerebral arteries  (433,434) | 1. Obstruction and stenosis of the anterior cerebral artery (433) |
|  |  | 2. Cerebral artery obstruction (434) |
| 66 | Late effects of cerebrovascular disease  (438) | 1. Late effects of cerebrovascular disease (438) |
| 67 | Other cerebrovascular diseases  (436,437) | 1. Diagnosis of acute cerebrovascular disease (436) |
|  |  | 2. Other and diagnosed cerebrovascular diseases (437) |
| 68 | Aortic aneurysm and dissection  (441) | 1. Aortic aneurysm and dissection (441) |
| 69 | Arterial embolism and thrombosis  (444) | 1. Arterial embolism and thrombosis (444) |
| 70 | Other arteries, small arteries and  capillary diseases  (442,443,446,447,448) | 1. Other aneurysms (442) |
|  |  | 2. Other peripheral vascular diseases (443) |
|  |  | 3. Nodular polyarteritis and related states (446) |
|  |  | 4. Other arterial and small arterial disorders (447) |
|  |  | 5. Capillary disease (448) |
| 71 | Bronchial pneumonia  (485) | 1. Bronchial pneumonia (485) |
| 72 | Pneumonia  (486) | 1. Pneumonia (486) |
| 73 | Other pneumonia  (480,481,482,483,484) | 1. Viral pneumonia (480) |
|  |  | 2. Pneumococcal pneumonia [Streptococcus pneumoniae pneumonia](481) |
|  |  | 3. Other bacterial pneumonia (482) |
|  |  | 4. Pneumonia caused by other specific pathogens (483) |
|  |  | 5. Pneumonia due to specific infectious diseases (484) |
| 74 | Chronic bronchitis  (491) | 1. Chronic bronchitis (491) |
| 75 | Pneumoconiosis and lung diseases caused by foreign substances  (500,501,502,503,504,  505,506,507,508) | 1. Pneumoconiosis in coal miners (500) |
|  |  | 2. Asbestosis (501) |
|  |  | 3. Other pneumoconiosis caused by silica or silica salts (502) |
|  |  | 4. Other inorganic dust caused by pneumoconiosis (503) |
|  |  | 5. Pulmonary disease caused by inhalation of other dust (504) |
|  |  | 6. Pneumoconiosis (505) |
|  |  | 7. Respiratory pathology due to chemical fumes and vapors (506) |
|  |  | 8. Pneumonia caused by solids and fluids (507) |
|  |  | 9. Respiratory disease caused by other foreign substances (508) |
| 76 | Other diseases of the respiratory system  (510,511,512,513,514,  515,516,517,518,519) | 1. Pulmonary pus (510) |
|  |  | 2. Pleural (rib) inflammation (511) |
|  |  | 3. Pneumothorax (512) |
|  |  | 4. Pulmonary and mediastinal ulcers(513) |
|  |  | 5. Pulmonary congestion and Shen sluggishness (514) |
|  |  | 6. Post-inflammatory pulmonary fibrosis (515) |
|  |  | 7. Other alveolar and alveolar wall pneumopathies (516) |
|  |  | 8. Specific diseases with pulmonary invasion (517) |
|  |  | 9. Other diseases of the lung (518) |
|  |  | 10. Other diseases of the respiratory system (519) |
| 77 | Chronic liver disease and cirrhosis  (571) | 1. Chronic liver disease and cirrhosis (571) |
| 78 | Hepatic abscess and the sequelae of chronic liver disease  (572) | 1. Liver pus ulcer and the sequelae of chronic liver disease (572) |
| 79 | Renal failure  (584,585,586) | 1. Acute renal failure (584) |
|  |  | 2. Chronic renal failure (585) |
|  |  | 3. Renal failure (586) |
| 80 | Other kidney diseases  (587,588,589) | 1. Renal sclerosis (587) |
|  |  | 2. Diseases caused by kidney malfunction (588) |
|  |  | 3. Small kidney of unknown origin(589) |
| 81 | Rheumatoid arthritis  (7140) | 1. Rheumatoid arthritis (7140) |
| 82 | Chronic post-rheumatic arthropathy  (7144) | 1. Chronic post-rheumatic arthropathy (7144) |
| 83 | Other inflammatory polyarthropathies  (7141,7142,7143,7148,7149) | 1. Felty's syndrome (7141) |
|  |  | 2. Other rheumatoid arthritis involving internal organs or the whole body (7142) |
|  |  | 3. Juvenile chronic polyarticular arthritis (7143) |
|  |  | 4. Other specific inflammatory polyarthropathies (7148) |
|  |  | 5. Inflammatory polyarthropathy (7149) |
| 84 | Systemic bone arthropathy  (7150) | 1. Bone arthropathy and related disorders (7150) |
| 85 | Primary localized bone arthropathy  (7151) | 1. Primary localized bone arthropathy (7151) |
| 86 | Progressive localized bone arthropathy  (7152) | 1. Recurrent localized bone arthropathy (7152) |
| 87 | Unspecified whole-body or localized  Osteoarthrosis  (7159) | 1. Unspecified systemic or localized osteoarthrosis (7159) |
| 88 | Ankylosing spondylitis and  other inflammatory spinal lesions  (720) | 1. Ankylosing spondylitis and other inflammatory spinal lesions (720) |
| 89 | Osteomyelitis, osteochondritis and  other infections affecting bone  (730) | 1. Osteomyelitis, periostitis and other infections invading bone (730) |
| 90 | Related to other specific diseases  metaplastic osteitis and bone lesions  (731) | 1. Metaplastic osteitis and bone lesions related to other specific diseases(731) |
| 91 | Anencephaly and similar malformations  (740) | 1. Anencephaly and similar malformations (740) |
| 92 | Spina bifida  (741) | 1. Spina bifida (741) |
| 93 | Others of the neurological system  Congenital malformations  (742) | 1. Other congenital malformations of the nervous system (742) |
| 94 | Congenital eye deformities  (743) | 1. Congenital eye malformation (743) |
| 95 | Congenital heart ball (embryo) and  Septal closure malformation  (745) | 1. Congenital malformation of the heart sphere (embryo) and septal closure (745) |
| 96 | Other congenital heart deformities  (746) | 1. Other congenital heart deformities (746) |
| 97 | Other congenital malformations of the circulatory system  (747) | 1. Other congenital malformations of the circulatory system (747) |
| 98 | Congenital respiratory system malformation  (748) | 1. Congenital respiratory system malformation (748) |
| 99 | Digestive system malformation  (749,750,751) | 1. Cleft palate and cleft lip (749) |
|  |  | 2. Other congenital anomalies of the upper gastrointestinal tract (750) |
|  |  | 3. Other congenital malformations of the digestive system (751) |
| 100 | Congenital malformations of the urinary tract  (753) | 1. Congenital malformations of the urinary tract (753) |
| 101 | Congenital musculoskeletal deformities  (754) | 1. Congenital musculoskeletal deformity (754) |
| 102 | Other congenital malformations of the extremities  (755) | 1. Other congenital malformations of the extremities (755) |
| 103 | Other congenital musculoskeletal deformities  (756) | 1. Other congenital musculoskeletal deformities (756) |
| 104 | Skull fracture  (800,801,802,803,804) | 1. Skull vault fracture (800) |
|  |  | 2. Skull base fracture (801) |
|  |  | 3. Facial fracture (802) |
|  |  | 4. Other skull fractures (803) |
|  |  | 5. Multiple fractures involving the skull or facial bones in combination with other bones (804) |
| 105 | Fracture of vertebral column, no mention of spinal cord injury  (805) | 1. Fracture of vertebral column, no mention of spinal cord injury (805) |
| 106 | Vertebral fracture, combined with spinal cord injury  (806) | 1. Vertebral fracture, combined with spinal cord injury (806) |
| 107 | Pelvic fracture  (808) | 1. Pelvic fracture (808) |
| 108 | Torso fracture  (809) | 1. Torso fracture (809) |
| 109 | Upper limb fracture  (810,811,812,813,814,  815,816,817,818,819) | 1. Clavicle fracture (810) |
|  |  | 2. Scaphoid fracture (811) |
|  |  | 3. Humerus fracture (812) |
|  |  | 4. Fracture of radius and ulna (813) |
|  |  | 5. Carpal fracture (814) |
|  |  | 6. Metacarpal fracture (815) |
|  |  | 7. One or more finger bone fractures (816) |
|  |  | 8. Multiple fractures of hand bones (817) |
|  |  | 9. Upper extremity fracture (818) |
|  |  | 10. Multiple fractures of upper extremities and upper extremities with ribs and sternum on both sides (819) |
| 110 | Lower limbs Fractures  (820,821,822,823,824,  825,826,827,828,829) | 1. Femoral neck fracture (820) |
|  |  | 2. Other fractures of the femur (821) |
|  |  | 3. Fracture of the bones of the pancreas (822) |
|  |  | 4. Fracture of tibia and fibula (823) |
|  |  | 5. Ankle fracture (824) |
|  |  | 6. Fracture of one or more tarsal bones and metatarsals (825) |
|  |  | 7. One or more toe fractures (826) |
|  |  | 8. Multiple fractures of the lower extremities and other fractures (827) |
|  |  | 9. Multiple fractures of both lower extremities, upper and lower extremities and lower extremities with ribs and sternum (828) |
|  |  | 10. Fractures (829) |
| 111 | Concussion  (850) | 1. Concussion (850) |
| 112 | Brain lacerations and contusions  (851) | 1. Brain laceration and contusion (851) |
| 113 | Post-injury subserosal membrane  subdural and epidural hemorrhage  (852) | 1. Subsarcoid, subdural and epidural hemorrhage after injury (852) |
| 114 | Intracranial hemorrhage after other injuries  (853) | 1. Intracranial hemorrhage after other injuries (853) |
| 115 | Other intracranial injuries  (854) | 1. Other intracranial injuries (854) |
| 116 | Visual nerve and nerve bundle damage  (950) | 1. Injury to the optic nerve and nerve bundle (950) |
| 117 | Other brain nerve injury  (951) | 1. Other brain nerve injury (951) |
| 118 | Spinal cord injury without significant spinal injury  (952) | 1. Spinal cord injury without obvious spinal injury (952) |
| 119 | Injury of the nerve root and spinal nerve plexus  (953) | 1. Injury to nerve roots and spinal nerve plexus (953) |
| 120 | Injury to the trunk nerves outside the shoulder and pelvis  (954) | 1. Injury to the trunk nerve outside the shoulder and pelvis (954) |
| 121 | Injuries to the shoulder and peripheral nerves of the upper extremities  (955) | 1. Injury to the shoulder and peripheral nerves of the upper extremities (955) |
| 122 | Peripheral nerve injury to the pelvis and lower extremities  (956) | 1. Pelvic and lower limb peripheral nerve injury (956) |
| 123 | Other nerve injuries  (957) | 1. Other nerve injury (957) |

**S3. Detailed Explanation of Index Date Design Based on a Nowcasting Framework**

In predictive modeling of disability, the index date served as the critical temporal anchor that defined the boundary between historical data used for feature construction and the outcome period. Establishing a clearly defined index date ensured that the model used only temporally valid information and prevented the inadvertent inclusion of future data, which would have compromised the validity and applicability of the model. The index date also simulated the real-world condition under which prediction would be made: a point in time when a decision-maker, such as a long-term care administrator or clinician, evaluated an individual’s health profile to estimate future risk.

This study adopted a nowcasting-based prediction framework, which aimed to detect whether an individual was currently experiencing—or was about to experience—disability using only data available prior to the index date. This approach differs from traditional long-term prognostic models that forecast outcomes over extended periods (e.g., 6 or 12 months ahead). Instead, nowcasting focuses on near-term risk identification and enables immediate action, making it well-suited for high-frequency surveillance and dynamic response scenarios. The index date, therefore, serves as the real-time reference point for generating risk predictions based on preceding medical history.

In our implementation, the definition of the index date differed by outcome group to reflect the observed data structure. For participants who developed disability during the observation period, the index date was defined as the date of disability diagnosis. For participants who did not develop disability, the index date was randomly assigned within their observation window, under the constraint that the individual had not yet developed disability and had at least three years of prior medical records available. In both groups, prediction features were constructed using disease-related information from the 3-year period preceding the index date, ensuring temporal alignment between input data and future outcomes while avoiding data leakage.

This framework enables consistent, batch-based nowcasting in real-world implementation. At any given time point, the model can be applied to all individuals with sufficient prior data to assess their current or imminent risk of disability. This structure supports repeated and scalable prediction cycles, facilitates real-time identification of high-risk individuals, and maintains methodological rigor throughout.

Figure S1 illustrated this design. Panel (A) showed how index dates were assigned and used during model training, with blue dashed lines indicating the 3-year look-back window prior to the index date. Red triangles denoted individuals who experienced a disability event, and blue circles indicated those who remained non-disabled. Panel (B) illustrated how the trained model would be applied in real-world nowcasting scenarios: all individuals were evaluated at a fixed prediction time point (e.g., December 10, 2020), using only their prior 3-year medical history to assess the likelihood of current or imminent disability. This approach ensured consistent and causal data flow, supported practical implementation, and aligned the training conditions with the intended deployment context of the model.

**
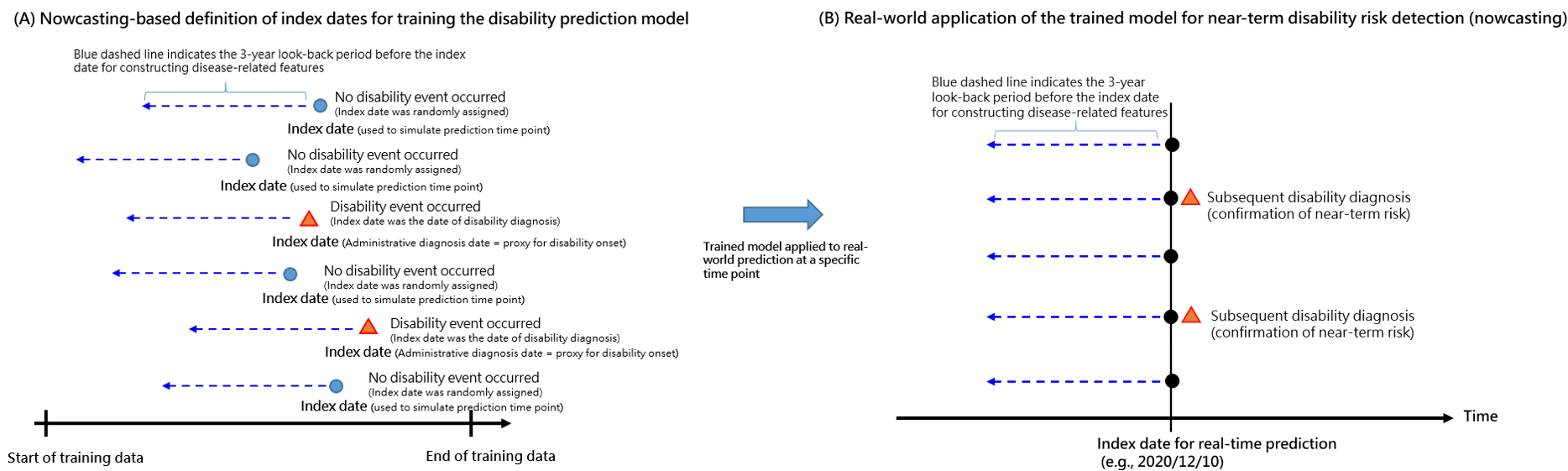
**

**Figure S1. Nowcasting framework for near-term disability risk detection: index date definition for model development and real-time application. Panel (A) illustrates the nowcasting-based training setup for the disability prediction model. For individuals who developed disability, the index date was defined as the administrative diagnosis date, serving as a proxy for the onset of functional decline. For those without recorded disability, index dates were randomly sampled from within the observation window to simulate population-wide, as-of-date surveillance conditions. All features were constructed from the 3-year look-back period prior to the index date (blue dashed lines), and no post-index information was used for model training—consistent with the nowcasting principle of using only pre-index data to estimate current risk. Panel (B) demonstrates the real-world deployment of the trained model for near-term disability risk detection. At a fixed prediction date (black dots, e.g., 2020/12/10), the model uses the prior 3-year medical history to generate risk scores for elderly individuals. Red triangles indicate individuals who were later diagnosed with disability. This setup reflects the model’s nowcasting objective: identifying individuals who are currently experiencing or imminently approaching disability, enabling timely outreach, triage, and resource allocation. This framework aligns with practical implementation needs for long-term care planning and policy response.**

**S4. The features sets selected by the feature-selection model**

Table S3. The features sets selected by the feature-selection model

| **Feature-selection model** | **Num.** | **Selected Features** |
| --- | --- | --- |
| FSM-  LightGBM | 36 | 1. Age  2. Sex  3. Hypertension disease  4. Heart failure  5. Intracranial hemorrhage  6. Occlusion and stenosis of cerebral arteries  7. Late effects of cerebrovascular disease  8. Pneumonia  9. Other pneumonia  10. Chronic bronchitis  11. Pneumoconiosis and lung diseases caused by foreign substances  12. Renal failure  13. Osteomyelitis, osteochondritis and other infections affecting bone  14. Skull fracture  15. Fracture of vertebral column, no mention of spinal cord injury  16. Vertebral fracture, combined with spinal cord injury  17. Pelvic fracture  18. Lower limbs fractures  19. Malignant neoplasm of digestive organs and peritoneum  20. Post-injury subserosal membrane subdural and epidural hemorrhage  21. Other intracranial injuries  22. Spinal cord injury without significant spinal injury  23. Malignant neoplasm of the respiratory tract and internal thoracic organs  24. Malignant neoplasm of genitourinary organs  25. Other malignant neoplasm and unspecified sites  26. Diabetes mellitus  27. Senile and presenile organic psychotic conditions  28. Other organic psychotic conditions (chronic)  29. Emotional psychosis (mania, depression, bipolar disorder)  30. Other psychiatric disorders  31. Psychotic disorders, personality disorders and other non-psychotic mental disorders  32. Alzheimer's disease  33. Other brain degeneration  34. Parkinson's disease  35. Hemiplegia and hemiparesis  36. Other paralytic syndromes |
| FSM-  Random Forest | 31 | 1. Age  2. Sex  3. Deafness (Loss of hearing)  4. Hypertension disease  5. Other forms of chronic ischemic heart disease  6. Heart failure  7. Intracranial hemorrhage  8. Occlusion and stenosis of cerebral arteries  9. Late effects of cerebrovascular disease  10. Other cerebrovascular diseases  11. Pneumonia  12. Chronic bronchitis  13. Other diseases of the respiratory system  14. Chronic liver disease and cirrhosis  15. Renal failure  16. Systemic bone arthropathy  17. Primary localized bone arthropathy  18. Unspecified whole-body or localized osteoarthrosis  19. Fracture of vertebral column, no mention of spinal cord injury  20. Upper limb fracture  21. Lower limbs Fractures  22. Unspecified tumor  23. Diabetes mellitus  24. Senile and presenile organic psychotic conditions  25. Other organic psychotic conditions (chronic)  26. Emotional psychosis (mania, depression, bipolar disorder)  27. Psychotic disorders, personality disorders and other non-psychotic mental disorders  28. Parkinson's disease  29. Other peripheral nervous system disorders  30. Other diseases of the retina  31. Glaucoma |
| FSM-  Decision Tree | 31 | 1. Age  2. Sex  3. Deafness (Loss of hearing)  4. Hypertension disease  5. Myocardial infarction  6. Other forms of chronic ischemic heart disease  7. Heart failure  8. Occlusion and stenosis of cerebral arteries  9. Late effects of cerebrovascular disease  10. Other cerebrovascular diseases  11. Other arteries, small arteries and capillary diseases  12. Bronchial pneumonia  13. Pneumonia  14. Chronic Bronchitis  15. Other diseases of the respiratory system  16. Chronic liver disease and cirrhosis  17. Renal failure  18. Systemic bone arthropathy  19. Primary localized bone arthropathy  20. Unspecified whole-body or localized osteoarthrosis  21. Upper limb fracture  22. Lower limbs fractures  23. Unspecified tumor  24. Diabetes mellitus  25. Senile and presenile organic psychotic conditions  26. Emotional psychosis (mania, depression, bipolar disorder)  27. Psychotic disorders, personality disorders and other non-psychotic mental disorders  28. Parkinson's disease  29. Other peripheral nervous system disorders  30. Other diseases of the retina  31. Glaucoma |
| FSM-  XGBoost | 25 | 1. Age  2. Hypertension disease  3. Heart failure  4. Intracranial hemorrhage  5. Occlusion and stenosis of cerebral arteries  6. Late effects of cerebrovascular disease  7. Arterial embolism and thrombosis  8. Pneumonia  9. Pneumoconiosis and lung diseases caused by foreign substances  10.Renal failure  11. Other congenital musculoskeletal deformities  12. Fracture of vertebral column, no mention of spinal cord injury  13. Vertebral fracture, combined with spinal cord injury  14. Pelvic fracture  15. Lower limbs fractures  16. Other intracranial injuries  17. Spinal cord injury without significant spinal injury  18. Other malignant neoplasm and unspecified sites  19. Diabetes mellitus  20. Senile and presenile organic psychotic conditions  21. Other organic psychotic conditions (chronic)  22. Other psychiatric disorders  23. Parkinson's disease  24. Hemiplegia and hemiparesis  25. Other paralytic syndromes |

**S5. The performance metrics of each predictive model based on 5-fold cross-validation using FSM-Random Forest, FSM-Decision Tree, and FSM-XGBoost feature-selected sets**

Table S4. The performance metrics of each predictive model based on 5-fold cross-validation using FSM-Random Forest, FSM-Decision Tree, and FSM-XGBoost feature-selected sets

Part A. The performance metrics of predictive model **in Training sets**

| **Feature-selected model** | **Predictive**  **Model** | **Accuracy** | **Sensitivity** | **Specificity** | **Balanced Accuracy** | **AUC** |
| --- | --- | --- | --- | --- | --- | --- |
| **FSM-Random Forest**  **(31 features)** | Random Forest | 0.7535 | 0.8243 | 0.7448 | 0.7845 | 0.8588 |
|  | XGBoost | 0.7566 | 0.8249 | 0.7481 | 0.7865 | 0.8588 |
|  | Logistic Regression | 0.7878 | 0.7593 | 0.7913 | 0.7753 | 0.8496 |
|  | Decision Tree | 0.7586 | 0.7397 | 0.7609 | 0.7503 | 0.8078 |
|  | LightGBM | 0.7543 | 0.8218 | 0.7460 | 0.7839 | 0.8551 |
| **FSM-Decision Tree**  **(31 features)** | Random Forest | 0.7503 | 0.8208 | 0.7416 | 0.7812 | 0.8549 |
|  | XGBoost | 0.7528 | 0.8199 | 0.7445 | 0.7822 | 0.8540 |
|  | Logistic Regression | 0.7847 | 0.7538 | 0.7885 | 0.7712 | 0.8455 |
|  | Decision Tree | 0.7596 | 0.7375 | 0.7623 | 0.7499 | 0.8076 |
|  | LightGBM | 0.7520 | 0.8151 | 0.7443 | 0.7797 | 0.8504 |
| **FSM-XGBoost**  **(25 features)** | Random Forest | 0.7537 | 0.8309 | 0.7442 | 0.7875 | 0.8618 |
|  | XGBoost | 0.7573 | 0.8330 | 0.7480 | 0.7905 | 0.8635 |
|  | Logistic Regression | 0.7942 | 0.7635 | 0.7979 | 0.7807 | 0.8575 |
|  | Decision Tree | 0.7538 | 0.7441 | 0.7550 | 0.7496 | 0.8084 |
|  | LightGBM | 0.7572 | 0.8317 | 0.7480 | 0.7899 | 0.8621 |

Part B. The performance metrics of predictive model **in Test sets**

| **Feature-selected model** | **Predictive**  **Model** | **Accuracy** | **Sensitivity** | **Specificity** | **Balanced Accuracy** | **AUC** |
| --- | --- | --- | --- | --- | --- | --- |
| **FSM-Random Forest**  **(31 features)** | Random Forest | 0.7523 | 0.8208 | 0.7438 | 0.7823 | 0.8528 |
|  | XGBoost | 0.7556 | 0.8208 | 0.7476 | 0.7842 | 0.8554 |
|  | Logistic Regression | 0.7878 | 0.7592 | 0.7913 | 0.7752 | 0.8496 |
|  | Decision Tree | 0.7586 | 0.7398 | 0.7609 | 0.7503 | 0.8077 |
|  | LightGBM | 0.7543 | 0.8215 | 0.7460 | 0.7837 | 0.8548 |
| **FSM-Decision Tree**  **(31 features)** | Random Forest | 0.7489 | 0.8163 | 0.7405 | 0.7784 | 0.8485 |
|  | XGBoost | 0.7518 | 0.8158 | 0.7439 | 0.7798 | 0.8505 |
|  | Logistic Regression | 0.7847 | 0.7537 | 0.7885 | 0.7711 | 0.8455 |
|  | Decision Tree | 0.7596 | 0.7371 | 0.7624 | 0.7497 | 0.8073 |
|  | LightGBM | 0.7520 | 0.8148 | 0.7442 | 0.7795 | 0.8502 |
| **FSM-XGBoost**  **(25 features)** | Random Forest | 0.7533 | 0.8294 | 0.7439 | 0.7866 | 0.8589 |
|  | XGBoost | 0.7571 | 0.8316 | 0.7479 | 0.7897 | 0.8622 |
|  | Logistic Regression | 0.7941 | 0.7635 | 0.7979 | 0.7807 | 0.8575 |
|  | Decision Tree | 0.7538 | 0.7441 | 0.7550 | 0.7496 | 0.8082 |
|  | LightGBM | 0.7572 | 0.8313 | 0.7480 | 0.7897 | 0.8620 |
